# From Risks to Roots: The Multifactorial Etiopathogenesis of Childhood Obesity

**DOI:** 10.3390/ijms27031527

**Published:** 2026-02-04

**Authors:** Vasile Valeriu Lupu, Alin Horatiu Nedelcu, Elena Jechel, Otilia Elena Frasinariu, Lorenza Forna, Ionela Daniela Morariu, Emil Anton, Dragos Catalin Ghica, Bogdan Puha, Cristina Maria Mihai, Silvia Fotea, Tatiana Chisnoiu, Ecaterina Grigore, Ancuta Lupu

**Affiliations:** 1 Grigore T. Popa University of Medicine and Pharmacy, 700115 Iasi, Romania; vasile.lupu@umfiasi.ro (V.V.L.); alin.nedelcu@umfiasi.ro (A.H.N.); lorenza.forna@umfiasi.ro (L.F.); ionela.morariu@umfiasi.ro (I.D.M.); emil.anton@umfiasi.ro (E.A.); dragos.ghica@yahoo.ro (D.C.G.); bogdan.puha@umfiasi.ro (B.P.); egrigore2002@gmail.com (E.G.); ancuta.ignat1@umfiasi.ro (A.L.); 2 Pediatrics, Faculty of Medicine, “Ovidius” University, 900470 Constanta, Romania; cristina2603@yahoo.com (C.M.M.); tatiana_ceafcu@yahoo.com (T.C.); 3 Faculty of Medicine and Pharmacy, “Dunarea de Jos” University of Galati, 800008 Galati, Romania; silvia_ghimpu@yahoo.com

**Keywords:** child, diet, disturbing exogenous factors, genetic predisposition, individualized pathophysiology and therapy, overweight

## Abstract

Pediatric obesity has shown a marked upward trend over the past decade, with a particularly significant impact in certain regions, to the extent that it is increasingly regarded as a global epidemic. The factors involved in its development and progression are highly diverse and complex. From genetic predisposition to the influence of epigenetic mechanisms, environmental exposures, nutritional patterns, psychosomatic factors, and endocrinological status, current evidence highlights multiple interacting pathways contributing to excessive weight gain in children. Although numerous studies have explored specific mechanisms and interventions, there remains a need for a comprehensive synthesis that integrates recent pathophysiological insights with practical clinical implications. This narrative review was undertaken to fill this gap by summarizing and analyzing the current literature on the mechanisms underlying pediatric obesity, emphasizing novel findings and evidence-based approaches. In light of recent advances in the field, this narrative review provides a comprehensive overview of the latest pathophysiological principles associated with childhood obesity, with particular emphasis on clinically relevant aspects. The review focuses on potential strategies to mitigate the impact of modifiable risk factors and highlights current trends in clinical research. The included studies were selected to cover the most relevant evidence on genetic, epigenetic, environmental, and psychosomatic determinants of pediatric obesity, providing a synthesis that informs both research and clinical practice. Its aim is to enhance the dissemination of knowledge regarding the underlying mechanisms involved in the development of pediatric obesity. In parallel, the review addresses evidence-based therapeutic approaches that may contribute to limiting the increasing incidence of the condition and its associated complications. Expanding the scope of scientifically grounded interventions may reduce obesity-related morbidity and substantially improve long-term outcomes in pediatric populations.

## 1. Introduction

The World Health Organization (WHO) defines obesity as “abnormal or excessive fat accumulation associated with a health risk”. Thus, childhood obesity is a serious medical condition that affects children and adolescents. This is classified using the body mass index (BMI), adapted depending on age and gender. Obesity is considered when there is an increase in the 95th percentile of BMI with a +1–2 standard deviations compared to the reference average for age and sex. The main cause of variability is attributed to physiological changes during the growth process [1,2].

From the perspective of comorbidities, excessive weight gain can negatively impact the health of young patients in both the medium and long term. This weight gain contributes to a heightened risk of developing metabolic, cardiovascular, neuro-psychological, gastroenterological, renal, respiratory, orthopedic, or dermatological disorders, pathologies considered to be of adults until recently. Comorbidities are now discovered as early as primary school, indicating that appropriate therapies can be quite effective. Additionally, childhood obesity induces psycho-social disturbances often manifested by low self-esteem, anxiety, or depression. The main risk factors (obesogenic factors) for childhood obesity include an inadequate diet rich in hypercaloric foods, a sedentary lifestyle, and family predisposition, as well as psychological factors (e.g., personal, parental, and family stress). Thus, the prevention and treatment of childhood obesity must represent a goal of modern medicine. In order to increase their efficiency in the medium and long term, it is important to emphasize that the two practices must be undertaken in an individualized manner, adapted to the characteristics of each individual patient [3,4,5,6,7].

Although numerous studies and reviews have examined childhood obesity, the majority have predominantly emphasized epidemiological trends or isolated mechanistic pathways, without systematically integrating contemporary pathophysiological insights into a clinically relevant framework. This narrative review is therefore designed to address this lacuna by synthesizing current evidence on genetic, epigenetic, environmental, and psychosomatic determinants, while concurrently highlighting their translational implications for prevention and clinical management.

Acknowledging the heightened scholarly interest in pediatric obesity alongside the ongoing imperative to refine diagnostic and therapeutic strategies, this work situates itself within the broader literature while emphasizing novel contributions. In this context, the review adopts a narrative approach to critically examine the key pathophysiological processes underpinning excess weight gain in children. Particular emphasis is placed on the interplay of genetic variability and epigenetic modulation in determining individual susceptibility and the trajectory of pediatric obesity. The final goal of the work is to increase the understanding of the pathological mechanisms underlying the onset of the pathology. In a secondary plan, we want to create a practical guide that brings together the main methods for counteracting exogenous disturbing factors, as well as the current directions and future premises of clinical research. In order to achieve our goal, we proceeded to integrate the most important international databases, using the following specific terms: “child”, “obesity”, “overweight”, “genetic factors”, “exogenous factors”, “diet”, “lifestyle”, and “psychosomatic disorders”, as well as their combinations. We did not impose period criteria regarding the screening of the literature, although we preferred the inclusion of recent articles (the last 10 years), without excluding the previous ones, of high reference in the field. Also, to reduce the risk of bias in reporting, we did not impose linguistic criteria.

## 2. Current Epidemiological Directions

It is challenging to provide an accurate estimate of the global impact of childhood obesity. This is mainly due to the great variability induced by the physiological stages that the body goes through during the growth and maturation process. Thus, in order to obtain a clear vision of the effect, it is necessary to subdivide the population into age groups and analyze them. The most recent reports note the incidence of childhood obesity as reaching high thresholds in countries with a high level of development. In parallel, a rapid upward trend can be noted in both low- and middle-income countries [1]. However, Pinhas-Hamiel O. et al. [8] conclude, through the comparative meta-analysis of studies published between 1960 and 2000, a marked increase in the rate of severe obesity in children in the 1980s, contrasting with a discrete slowdown in 2000. The *World Obesity Atlas* 2023 estimates that pediatric obesity affects 2.6 million children under the age of 5 and up to 175 million children between the ages of 5 and 19. The prevalence of childhood obesity has skyrocketed in recent decades, and within less than 30 years, it could impact up to 25% of children. As the age range increases, the gender ratio tilts in favor of females [9,10]. This trend seems to be variable depending on the demographic data. For example, it is mainly observed in the countries of Sub-Saharan Africa and Oceania, as well as in other middle-income countries. In contrast, obesity was reported more frequently in boys in all high-income countries and in all East and Southeast Asian countries [11,12]. The increased predisposition of girls towards the development of excess weight can be partially attributed to hormonal variability [13].

In the global context previously outlined, the increase in understanding regarding the main factors that drive the amplification of obesity among pediatric patients is vital. Through this, clinicians can lay the foundations for effective, multidisciplinary prevention, detection, and management programs for children at risk, in order to ultimately improve their quality of life [14,15]. Recently, Leung AKC. et al. [16] have emphasized the fact that, once diagnosed, childhood obesity frequently responds poorly and incompletely to standard therapeutic measures. Therefore, the efforts of practitioners must be focused rather on prevention by promoting a healthy, active lifestyle. In this direction, Berleze et al. [17] draw attention to the importance of promoting motor development and increasing self-esteem, commitment, and joy found in physical activity among overweight children and children with obesity. These desires come together under the umbrella of the concept of “Mastery Motivational Climates”—the awareness of the fact that the effort–results relationship is bidirectional. The results of the study demonstrated that overweight children and children with obesity need an appropriate climate to feel motivated and involved. Once this is outlined, they manage to reduce sedentary activities (e.g., time spent in front of the TV) and compensate with dynamic activities.

Next, Aceves-Martins et al. [18] argue that interventions should not be limited to educational activities. In order to increase efficiency and reliability over time, they should include different methods of behavioral modulation, including familiar characteristics and the social environment [18]. In agreement with them, Tester JM. et al. [19] demonstrate that experiencing food insecurity in the family imprints the trajectory of the weight curve among children with obesity. A challenge not to be neglected is represented by medically complex or premature children. They are more prone to being overweight compared to children without comorbidities, and at the same time are more difficult to manage [20,21,22].

## 3. Pathophysiological Considerations

Broadly speaking, obesity is known to represent weight gain partially attributed to high caloric intake, the preferential use of processed, energy-dense, and nutritionally poor foods that induce increased insulin release and fat storage [11]. In a more complex vision, the causality of childhood obesity can be subdivided into prenatal, perinatal, and postnatal factors. If in the first category we find mainly the factors related to maternal health, after birth, aspects such as the type of birth, birth weight, and its variations during childhood, breastfeeding, the moment of diversification, the formation of food preferences, the influence of the environment, or socio-economic inequalities become important in dictating the risk of overweight. Critical windows of biological–behavioral plasticity, considered favorable moments for intervention to change the patient’s evolutionary course, are thus outlined. Genetic factors represent an essential, timeless variable, able to intervene in various moments of development [15,23,24,25]. All of them make up an “obese” environment. Obesity is therefore described by the interrelation of a mixture of genetic and epigenetic factors, behavioral, socio-cultural, and environmental risk models that compete to induce imbalances in the two body-weight-regulation systems (energy homeostasis and cognitive–emotional control) [11,26].

**Figure 1:**
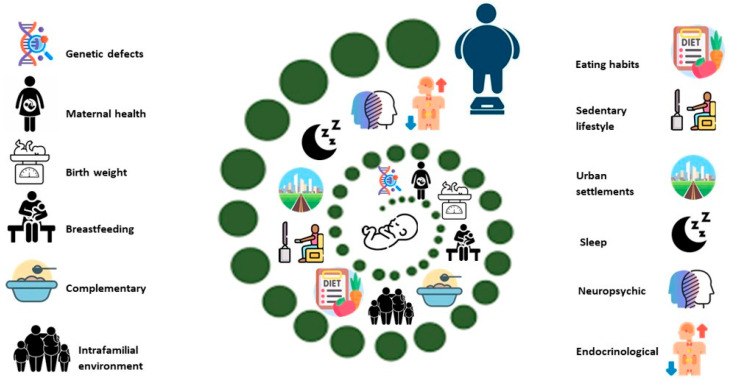
Pathophysiological conditions that potentiate the appearance of childhood obesity.

A particular type of obesity is represented by obesity with central predisposition. This is accompanied by a normal weight. Although its cardiometabolic implications are known, the investigation of precipitating factors is modestly found in the literature. In this sense, Ntimana CB. et al. [27] notes, in addition to the previously mentioned, the determinant of gender, the physiological moment of development (relative to puberty), and the age of the parents as decisive factors in increasing the risk of developing central obesity in children. The importance of adequate screening for central obesity among children lies in the increased risk of omission due to normal BMI values [28]. The most reliable anthropometric indices for predicting general and central obesity in preschool children seem to be the body mass index and neck circumference [29]. In this sense, Figure 1 schematically illustrates the main factors that shape the risk of developing obesity at a pediatric age.

### 3.1. Role of Genetics

The role of genetics in defining individual anthropometric characteristics was best exposed by observing children from twin pregnancies. There is therefore a substantial correlation between parents and the development model of their biological offspring [30,31]. These data were also validated in an interfamilial context, independent of the twin brother [32]. Therefore, genetic inclination cannot be the only explanation for the upward trend in obesity prevalence. Thus, the mutual interrelationship among the genetic factors dictating the predisposition to excess weight, the epigenetic component, and psycho-somatic or exogenous variables remains open to research. The importance of these studies is valuable from the perspective of the discovery of possible useful biomarkers for preventive medicine and early clinical management of the condition [33,34,35,36,37].

Regarding genetic predisposition, there are few reports on the implications of single-gene defects (insufficiency or disorders) in dictating pediatric obesity, particularly monogenic obesity. This situation occurs mainly in cases of extreme obesity or in isolated groups of people. The best-known genes involved in the etiopathogenesis of obesity are leptin (*LEP*), leptin receptor (*LEPR*), proopiomelanocortin (*POMC*), prohormone convertase 1 (*PCSK1*), melanocortin receptor 4 (*MC4R*), homologue 1 (*SIM1*), brain-derived neurotrophic factor (*BDNF*), and the neurotrophic receptor tyrosine kinase type-2 gene (*NTRK2*). Other genes described are adiponectin, C1Q and collagen domain containing (*ADIPOQ*), adrenoceptor beta 2 and 3 (*ADRB2*, *ADRB3*), insulin receptor substrate 1 (*IRS1*), neuropeptide Y (*NPY*), peroxisome proliferator-activated receptor gamma (*PPARG*), peroxisome proliferator-activated receptor gamma coactivator 1 alpha and beta (*PPARGC1A*, *PPARGC1B*), protein tyrosine phosphatasen non-receptor type 1 (*PTPN1*), solute carrier family 22 member 1 and 4 (*SLC22A1*, *SLC2A4*), sterol regulatory element binding transcription factor 1 (*SREBF1*), and uncoupling protein 1 (*UCP1*). In most cases, however, obesity seems to be the result of the interactions among several genes (polygenic obesity) and various environmental factors, thereby potentiating the obese phenotype characteristic of non-syndromic excess weight [15,38,39]. Association studies at the level of the human genome have identified numerous loci associated with the regulation of BMI or waist–hip ratio adjusted for BMI, with similar findings for the adult and pediatric populations. Additionally, there are over 300 single-nucleotide polymorphisms (SNPs) whose functionality is still being researched from the perspective of how they influence the phenotype. Their study reiterates the fact that BMI is modulated mainly by the hypothalamic control of energy intake. However, lifestyle is a modulator of the risk of developing obesity among patients with genetic predisposition. Discoveries in the field can represent new foundations of individualized preventive and curative management of obesity [40,41,42].

The fat mass, obesity associated gene (*FTO*), and the melanocortin 4 receptor (*MC4R*) are the most extensively researched genes in determining anthropometric features, as well as the degree and timing of obesity and its related comorbidities. It is known about *FTO* that its SNPs can induce changes in the expression of other genes, with repercussions for the brain, pancreas, and adipocyte balance. At the same time, *FTO* has been shown to be involved in increasing the risk of comorbidities associated with obesity—metabolic, hepatic, and cardiovascular [40,43,44]. With regard to *MC4R* variants (mainly c.496G > A), Nalbantoğlu Ö. et al. [45] note that they are the most common genetic cause of early-onset monogenic obesity. In agreement with them, Serra-Juhé C. et al. [46] support the clinical utility of genetic testing to identify susceptible patients for targeted therapeutic intervention. Next, we note that *FTO* seems to play a minor role in modulating the variability of responses to interventions against pediatric obesity. However, the impact of the accumulation of several predisposing genes on individual therapeutic response remains under investigation [47]. Future research efforts should focus on the development of effective inhibitors (natural or pharmacological) of *FTO* expression [46]. Next, Table 1 summarizes the main characteristics of the most frequent genes involved in childhood obesity.

In practical terms, genetics intervenes in body weight dynamics by regulating food intake, energy consumption, and adipogenesis. Depending on the individual genetic defects, two forms of obesity are distinguished: syndromic and non-syndromic. Compared to the latter, presented above, syndromic obesity targets the main chromosomal abnormalities that either directly associate obesity within the diagnostic criteria, or indirectly predispose to an increase in its prevalence compared to the general population. Currently, more than 79 such syndromes are known (e.g., Prader–Willi syndrome, Down syndrome, Bardet–Biedl syndrome, fragile X syndrome, Alstrom syndrome, Cornelia de Lange syndrome), of which 55 include obesity among the clinical manifestations, and in 24 of them, being overweight is more frequent compared to the control group [38]. Finally, the acquisition of basic genetic knowledge is vital for the practicing physician, as an overlap between genes and pathways found in both syndromic childhood obesity and non-syndromic polygenic obesity is mentioned in the current medical literature [48].

### 3.2. Implications of Environmental Factors

As we mentioned before, genetics cannot represent the only variable predisposing to overweight. The socio-economic-cultural status and life habits imposed on a fragile genetic background can adversely affect the prevalence of obesity from childhood, depending on the demographic characteristics and individual aspects of the patients [49,50,51,52]. As recently noted by Gutiérrez-González E. et al. [53], a poor socio-economic status in the family is associated with unhealthy eating habits and sedentary behavior (especially in the case of girls). The common point of the two seems to be represented by a prolonged time spent by children/adolescents in front of screens (television, laptop, phone). It is estimated that the risk of developing overweight/obesity increases proportionally with the number of hours the child is exposed to screens, regardless of gender. The mechanisms involved are the decrease in the time allocated to physical activities and effective rest, the decrease in social interactions, and the increase in overeating based on snacks rich in fats/sugars [54]. Also, children who live in the same household with an older sibling who is overweight or obese have an increased predisposition of up to four times to develop excess weight. The considerations are based on the hypothesis of “common family environments”, in which participants share similar diets and participate in the same type of physical activity [55].

Next, parental control seems to show a correlation with BMI. This was objectified by Ji M. et al. [56] who studied 631 pairs of same-sex monozygotic twins. The authors found that, controlling for the genetic component, environmental characteristics, and body-weight level at the beginning of the research, the twin with harsher parents in terms of communication had a lower BMI than his co-twin. However, further studies are needed to estimate the size of the effect between parent–child communication and predisposition to obesity, alcoholism, or smoking.

Consequently, current efforts must focus on redressing social inequalities among the high-risk pediatric population. In parallel, we must consider interventions targeting family aggregates that share and transgenerationally perpetuate obesogenic features. In this sense, we aim to promote an active lifestyle both at school and outside of class, creating communities with healthy principles (preference for walking/cycling, practicing outdoor sports, predominantly using mixed food stores at the expense of conventional supermarkets), supported by education and continuous nutritional intervention [57,58]. The initiative is in agreement with the WHO directives regarding the surveillance of childhood obesity in Europe [59]. Therefore, in order to be effective, the measures must benefit from common community support. The final goal is mainly a preventive one, to which is added in the second plan the need to reduce the burden faced by parents who have children with severe obesity [60].

In addition to those previously presented, the quality of the environment proved to be an important variable in dictating the risk of childhood and intrafamilial obesity [61]. In the same direction, characteristics such as increased pollution, heavy traffic, and noise encountered in urban settlements are associated with an increased obesogenic risk, according to Bont J. et al. [62]. Similar findings were obtained by Oktaviani S. et al. [63]. Last but not least, sleep is an important ally in childhood. Sleep deprivation for various reasons is associated with excess weight. The pathophysiological mechanism incriminated is based on disturbances in metabolism (e.g., decreased sensitivity to insulin) and hormonal balance that influence appetite, the desire to eat, and food preferences [64]. The data were also certified by Sari GN. et al. [65]. They argue that a short duration of nighttime sleep among girls aged 2.5 years was positively correlated with the risk of obesity at age 5.5 years.

### 3.3. Footprint of the Dietary Component

Eating habits represent another variable worth exploring in pediatric obesity. Their importance is bivalent both from the perspective of inducing and maintaining excess weight and regarding the potentiation of the risk of nutritional deficits, when the child is non-compliant (e.g., inadequate intake of vegetables, fruits, and dairy products, excessive consumption of caloric snacks) [66]. In this direction, Mameli C. et al. [67] identify three important steps (prenatal period, breastfeeding vs. formula feeding, and complementary diet) in dictating the child’s predisposition to obesity. Thus, the theory of “the first 1000 days of life” comes into focus, which, similar to the importance of modulating the intestinal microbiota, appears to constitute a critical window for the action of the main disruptive factors that induce the development of childhood obesity [67,68].

In relation to the chosen topic, our primary focus is the perinatal period. In this aspect, even beyond the intrauterine period, maternal metabolic health has a strong influence on dictating the trajectory of the newborn through breastfeeding. Disturbances in maternal homeostasis can modulate the composition of immune components in breast milk (e.g., immunoglobulins, lactoferrin, leptin, ghrelin, adiponectin, C-reactive protein, growth factors, extracellular vesicles, and lymphocytes). Thus, the risk–benefit balance regarding breastfeeding of children from mothers with obesity, as well as the optimal period for this, remains a topic of interest in research [69]. In the absence of pathological changes in the maternal organism, the international recommendations in force support a balanced maternal diet rich in nutrients, similar to the prenatal period. The breastfeeding program aims to achieve a gradual increase in weight defined by generally accepted growth standards, avoiding excessive or insufficient feeding. Exclusive breastfeeding or mixed, protein-balanced feeding (where the former is not possible) are known to have implications in reducing the risk of overweight and subsequent obesity by 12–14%. The protective capacity proved to be directly proportional to the period in which breastfeeding was practiced, being more effective for a duration of 6 months or more [70,71,72]. In agreement with the above, previously, Sandoval Jurado L. et al. [73] demonstrated that exclusive breastfeeding for less than three months was associated up to four times more frequently with childhood obesity. The protective effect of breastfeeding on obesity, but also the size of the effect, proportional to the duration of breastfeeding, was later confirmed by Ma J. et al. and Qiao J. et al. [74,75] through meta-analyses of data. Additionally, Hering A. et al. [76] draw attention to a positive correlation between breastfeeding history and average sleep duration, doubled by a negative correlation between the latter and the risk of obesity.

Next, the same previous recommendations claim that complementary feeding should be optimally introduced in the window of 17–26 weeks. This represents a crucial moment in the infant’s life, both in relation to rapid changes in nutritional needs and for the formation of taste preferences and eating habits [70,77]. The introduction of complementary food before the mentioned period proved to be directly associated with the child’s adiposity. In parallel, the limitation of foods/drinks rich in sugar and the hyper-protein diet in early childhood must be taken into account [70,78]. The development of complementary feeding methods is undergoing continuous research. In this sense, the practice of baby-led weaning has been shown to be a beneficial method to obtain a decrease in fussiness, a greater pleasure of feeding, and increased satiety, conferring at the same time a reduced risk of obesity [79]. Furthermore, responsive feeding has been shown to be effective in promoting adequate infant growth [80]. The current study directions, therefore, focus on delimiting the best feeding practices (receptive/non-receptive/traditional feeding). Among the three, children from families that adopted receptive feeding as a form of complementary feeding also had lower BMIs in early childhood [81].

After early childhood, the feeding of older children and adolescents must follow clear principles to avoid excess weight. From a wider perspective, these are represented by avoiding the consumption of high-calorie, nutrient-poor foods (e.g., sugar-sweetened drinks, sports drinks, soft drinks, most “fast food”) and encouraging the consumption of dietary fiber, vegetables, and seasonal whole fruits rather than fruit juices [82]. However, additional studies are needed to correctly assess the risk–benefit ratio of snacks between meals. From a caloric point of view, they provide an intake of up to 565 kcal/day. Nutritionally, snacks provide an important amount of fat and protein, as well as a large part of nutrients (e.g., vitamin C, D, E, B12, sodium, potassium, magnesium, iron, calcium, and folic acid) [83]. Their consumption has proven to be vital in a wide range of chronic diseases, starting from metabolic disturbances (e.g., diabetes, obesity) and ending with atopic diseases. Their role resides mainly in their strong antioxidant capacity, necessary to counteract the damage induced by oxidative stress specific to these long-term inflammatory pathologies [84]. For an easier distinction, Liberali R. et al. [85] divide food components into potentially obesogenic foods (e.g., fatty cheeses, sugary drinks, processed foods, fast food, candy, snacks, cakes, animal products, whole milk, and refined grains) and healthy foods (e.g., fruits, vegetables, whole grains, fish, nuts, legumes, and yogurt). Also, the consumption of ultra-processed foods is associated with an increase in energy density and the amount of free sugars, in contrast to a decrease in fiber [86]. R these, De Amicis R. et al. [87] concluded that, consumed in constant quantities, they can influence the nutritional status and body composition of children and adolescents over time.

An important ally in reducing pediatric obesity seems to be a balanced breakfast, based on dairy products, cereals, and fruits. In addition, regular breakfast helps combat nutritional deficiencies (e.g., vitamin D), important components in the pathogenesis of obesity and related disorders [88,89,90]. Also, the Mediterranean diet has been reported to have a positive impact on improving anthropometric indicators and pediatric obesity [91]. At the opposite pole, Jiang J. et al. [92] have recently demonstrated, through the meta-analysis of 60 studies, that the distance between fast-food or other unhealthy food restaurants and the residence can be correlated with the weight of children/adolescents. Previously, Potvin Kent M. et al. [93] drew attention to food marketing towards teenagers using social applications. They highlight the need to formulate regulations aimed at restricting the sale of unhealthy foods on social media. Last but not least, family meals have been shown to be a protective factor in combating overweight and obesity among children and adolescents. Consequently, they must be encouraged, especially in this age group [94].

### 3.4. Psycho-Somatic Disturbances

The link between mental disorders and body composition has been shown to be double-edged. Thus, both externalizing and internalizing symptoms appear to be associated with obesity. As an example, we observe a correlation between low inhibitory control capacity/impulsivity and high body weight. The deficit is implicated in the obesity–attention deficit/hyperactivity disorder (ADHD) association [95,96,97]. Additionally, it is noted in the specialized literature that psychopharmacological medication (e.g., psychostimulants, neuroleptics, antidepressants) has the potential to influence weight and body characteristics [95]. Also, severe hyperphagia with/without impulsivity is one of the most frequent causes of early childhood obesity (under the age of six). It is important to note that this can be effectively evaluated using Dykens’ and Impulsivity questions [98]. In the same direction, Grazuleviciene R. et al. [99] draw attention to the fact that an unfavorable psychosocial environment and a pathological mother–child relationship can represent factors predicting overweight/obesity in children. Overweight and obesity predispose to disorders regarding body perception and low self-esteem [100,101]. The most vulnerable population categories emotionally affected by overweight/obesity are young children, girls, and those who cannot exercise adequate control over food [102].

Stressors can induce direct physiological changes by modulating various regulatory pathways. Among these, we note mainly the activation of the hypothalamic–pituitary–adrenal (HPA) axis and the sympathetic nervous system, as well as other systems. Following activation, physiological changes occur in the cascade at the systemic level, including increases in serum cortisol and glucose, or the potentiation of the pro-inflammatory status. Also, HPA balance appears to be interconnected with sleep disturbances, another important component previously described in pediatric obesity [51,103,104]. In particular, cortisol acts by binding to specific receptors in the peripheral and central nervous system. Its effects materialize by mobilizing and redistributing energy stores to maintain homeostasis until the stressor disappears. In this sense, the excretion rate of free cortisol from urine was positively correlated with BMI and central adiposity. Chronic action influences gluconeogenesis, glycogenolysis, lipolysis, insulin resistance, reproductive function, and eating behavior. Dysfunction of the HPA axis can potentiate the risk of metabolic and affective disorders. Paraclinically, in response to stress, the body develops new “reference points”. Additionally, there is an increase in the risk of chronic diseases, including obesity or metabolic syndrome. Evolutionarily, one of the causes of the increased sensitivity of adolescents to the action of stress is attributed to the successions in the development of brain regions (the limbic system involved in motivation, satisfaction, and reward develops much faster than the cortical areas responsible for inhibitory control) [103,105,106].

### 3.5. Endocrinological Factors

Fewer than 1% of children and adolescents experience endocrinological disorders (e.g., endogenous/exogenous glucocorticoid excess, hypothyroidism, growth hormone deficiency, pseudohypoparathyroidism type 1a, hypogonadism, insulinoma, hypothalamic obesity, polycystic ovary syndrome) accompanied by secondary obesity. The developmental pattern of these children is represented by poor linear growth, short stature, and/or hypogonadism [107,108].

The way in which BMI increases in these conditions is varied, depending on the action of the hormones involved. Among the most well-known examples, we note that in hypothyroidism there is a decrease in the basic metabolism with a decrease in energy consumption, water retention, and infiltration of the subcutaneous cellular tissue with mucopolysaccharides. In Cushing’s or other forms of hypercortisolism, there is an increase in lipogenesis in the face and abdominal tissue (central obesity), accompanied by hyperphagia. The pathophysiology of obesity accompanying growth hormone (GH) deficiency has not been fully understood. However, GH is known to play a significant role in energy metabolism, body composition, bone mineral density, lipid metabolism, and cardiovascular function. GH deficiency contributes to decreased muscle mass and the deposition of truncal fat. Affected children, although they appear obese, usually have normal BMI values. Last but not least, damage to the hypothalamus, especially in the ventromedial region, leads to manifestations such as hyperphagia, decreased resting metabolic rate, hypomotility, insomnia, and autonomic imbalance, also inducing hormonal disorders (GH, gonadotropins, thyroid-stimulating hormone). Also, the hypothalamic nuclei regulate appetite and energy consumption through neuropeptide control [108,109]. In summary, key findings to guide the clinician in considering pediatric endocrinological obesity include childhood onset, satiety, poor linear growth, dysmorphic features, and cognitive dysfunction [110]. However, the current findings support that, except for hypothyroidism, screening for obesity-determining endocrinopathies should be performed judiciously. Currently, most tests are not recommended in the absence of clinical characteristics of endocrinological syndromes, and hormonal treatment is rarely necessary. We also note that effective weight loss has the potential to contribute to the correction of endocrinological dysfunctions [111].

## 4. Practical Directions

The main concern regarding childhood obesity is attributed to the morbidity with which it is associated in the medium and long term. According to Llewellyn A. et al. [112], reducing pediatric obesity does not seem to substantially reduce the burden of morbidity in future adults with obesity. However, the risk of childhood obesity being perpetuated later in adult life cannot be neglected. In this sense, complex interventions aimed at modifiable pre-conception, prenatal, perinatal, and early variables must represent a mandatory goal in the prevention of overweight at preschool age [113]. In order to increase the efficiency of the results, the individualized therapy of the patient must be put into practice with the help of a multidisciplinary team. Another important point is the choice of the optimal moment for the intervention. It seems that, with advancing age, the effect of lifestyle changes on health parameters decreases [114]. Therefore, an overview of the main vulnerable points in the emergence and maintenance of pediatric obesity is summarized in Table 2. In agreement with what has been stated, we focus our attention on the following: drawing the main, important lines of the prophylactic and adjuvant, personalized, non-pharmacological management of the overweight pediatric patient.

### 4.1. Means of Prevention and Reduction of the Impact of Risk Factors

Therefore, obesity in childhood and adolescence is associated with a series of medical and psychological complications, increasing the predisposition to type-2 diabetes, hypertension, dyslipidemia, and non-alcoholic steatohepatitis. All these comorbidities can increase the chance of hospitalization in the pediatric intensive care unit when the general condition deteriorates, thus negatively affecting the patient’s evolution by adding to the risk of developing healthcare-associated infections. In order to reduce the risk of overweight at the pediatric age, we must consider management based on life cycles: early feeding of infants, parental food patterns, early gain of adiposity, socio-economic status, racial affiliation, and pathological antecedents of the child (especially the endocrinological ones) and associated drug therapy [115,116,117,118]. In this sense, Strzelecka I. et al. [119] argue that reducing the consumption of sweets, coupled with increasing the nutritional awareness of parents and children, can be important factors in preventing the development of childhood obesity. Current studies show an insufficient level of primary medical care aimed at overweight children. Consequently, the creation and implementation of BMI monitoring programs for children and adolescents with risk factors or confirmed diagnosis is encouraged. At the same time, changing the environment offers opportunities for the development of healthier behaviors. Therefore, obesity prevention programs should combine person-based and environmental approaches [120,121]. In parallel, Bedell D. et al. [122] note the importance of encouraging physical activity in the age group 3–6 years. They note that decreased physical activity correlates more closely with preschool overweight/obesity than with caloric intake. However, Zhao L. et al. [123] note that physical exercises combined with diet are more effective than any of the measures taken individually. Regarding pharmacological and surgical therapy, it is known that anti-obesity drugs have a limited role in childhood, not being recommended for small children. Also, bariatric surgery is reserved for adolescents with morbid obesity, with data on its long-term safety being limited in this age group [118].

The progress of research on overweight children leads us to the approach of early genetic diagnosis and specialized, individualized multidisciplinary care. The detection of a genetic cause of obesity must be followed by the institution of an intensive intervention that includes lifestyle modification, targeted pharmacological treatment, and bariatric surgery (in cases that meet the criteria). The final goal is to prevent the development of complications (e.g., cardiometabolic, psychosocial) and reduce the stigmatization of patients and families. At the same time, the medical literature takes into account the implications of the intestinal microbiota, a bioactive system in constant change, in increasing the risk of developing multiple diseases—autoimmune, inflammatory, atopic—but also potentiating the systemic decline from chronic diseases. All of these seem to be modulated by means of the intestines–vital organs axes [124,125,126]. Regarding pediatric obesity, current studies attest to a change in the intestinal microbiota (dysbiosis) both in genetic and non-genetic etiology [127].

In agreement with Maya-Lucas O. et al. [128], knowledge of the dysbiotic pattern is important from the perspective of the possibility of stratifying individuals based on their phenotype. However, the ethnic variability of the microbiome cannot be neglected [129]. Another variable proposed as contributing to disturbance of the intestinal microbiota is exposure to antibiotics during the perinatal period. This situation seems to tilt the phenotypic balance of the host towards a predisposition to obesity, beyond the interruption of antibiotherapy and the recovery of the intestinal microenvironment [130]. In practical terms, dietary modulation through the consumption of non-digestible, fermentable carbohydrates has been shown to have a positive effect in promoting beneficial bacteria and reducing those producing toxins. This mechanism contributes to the mitigation of metabolic damage associated with obesity, regardless of the primary trigger [127]. Additionally, targeting gut microbiota and metabolites (e.g., short-chain fatty acids, medium-chain fatty acids, amino acids, amines, and bile acids) may represent a promising strategy for reducing overweight in children [131]. In this sense, the intake of Lactobacillus (mainly due to its metabolite, phenyllactic acid) and biotic components (prebiotics, probiotics, symbiotics) represents a possible direction of adjuvant therapy. Their role is both prophylactic (e.g., regulates intestinal lipid metabolism) and adjuvant in pediatric obesity [132,133].

Last but not least, the psychosocial component must be carefully monitored from two different perspectives. We refer here both to the prejudices that come with weight gain, as well as to the possible adverse effects of psychoactive substances (e.g., atypical antipsychotics) on body weight [134,135]. Thus, the main psychosocial barriers encountered by children and parents of overweight children were interpersonal differences regarding the implementation of dietary changes, protecting self-esteem, prejudices related to excessive weight, lack of clinicians with experience in obesity, and lack of access to weight-management services [136,137]. We believe that finding ways to address these inadvertences could represent an important step in the management of childhood obesity.

### 4.2. Current Clinical Research

Childhood obesity continues to be a subject of extensive investigation. Among the most recent studies in the field, we note the attempt to define the critical age period and the individual variables that mark the increase in the risk of excess weight later. In this direction, Mellado Peña F. et al. [138] note that an age of 4 years, the male sex, the poor educational status of the mother, and living together with the grandparents are the main attributes that indicate the right moment for the primary, prophylactic medical intervention. Additionally, Mondal PK. et al. [139] imagine several models for predicting obesity risk based on information gathered from a single/multiple doctor visits. At the same time, we do not lose sight of the findings issued by Zhang Y. et al. [140]. The authors argue that a higher height in early childhood (4 years), compared to children of the same age and sex, can be an additional indication of the predisposition to obesity and subsequent pubertal disorders.

Another important aspect is represented by the impact of chronic low-grade inflammation associated with overweight mothers during pregnancy. The immune–inflammatory pathways and the pathophysiological mechanisms involved in the potentiation of risk in offspring, depending on gender, remain to be elucidated [141]. From a clinical point of view, the analysis of data from the literature attests to the harmful effect of overweight/prenatal obesity in compromising neurodevelopment. The main mechanisms incriminated in this correlation are maternal and fetal inflammation, changes in the microbiome, epigenetic changes in neurotrophic genes, and impaired dopaminergic and serotonergic signaling [142]. Thus, Mine TH. et al. and Sanchez C. et al. [143,144] argue that obesity before pregnancy increases the risk of attention deficit hyperactivity disorder, autism spectrum disorder, developmental delay, and emotional/behavioral disturbances (e.g., anxiety/depression, aggressive behavior). Consequently, very severe maternal prenatal obesity remains a significant predictor of neuropsychiatric disorders in offspring, independent of demographic, prenatal factors, and concurrent maternal psychiatric conditions.

Implications of maternal weight have also been suggested in the case of pediatric cardiovascular diseases. In this situation, we identify a different structure and functionality of children from overweight mothers compared to the control groups. The most important observations were the reduction of systolic function, with its persistence during childhood, and a thicker interventricular septum [145]. Similar data were objectified by den Harink T. et al. [146]. The authors encourage mothers to change their lifestyle during the preconception period. Its advantages arise from improved cardiac structure and function in the descendants, marked by a thinner interventricular septum, lower left ventricular mass, and improved Doppler velocities.

To integrate recent molecular insights and scientific advances in the understanding of pediatric obesity, Table 3 summarizes the most relevant genetic, epigenetic, and molecular regulatory findings, including gene–environment interactions and markers of susceptibility to obesity phenotypes. This review complements the clinical observations mentioned above, providing a link between molecular mechanisms and effects observed in offspring, and highlights current research directions in the field.

## 5. Conclusions

In conclusion, regardless of the analytical approach or conceptual framework considered, the development of obesity forms a vicious cycle that is hard to break. In our manuscript, we explored the key prenatal, perinatal, and postnatal risk factors that affect children’s ability to maintain an optimal weight. Obesity, therefore, represents a combination of intrauterine variables, genetic susceptibility, the influence of environmental factors, parental models regarding physical activity, dietary preferences and objections, and behavioral characteristics. We also considered the impact of psychological and endocrinological factors. While a direct link between pediatric obesity and systemic decline in adulthood could not be definitively established, the wide range of comorbidities affecting overweight children cannot be overlooked.

In order to reduce the incidence and the impact of obesity at a pediatric age, global efforts must focus on the outline of a unanimous obesity prevention and screening strategy, directed in an individualized way depending on the risk factors and possible etiopathogenesis. Additionally, once the predisposition to obesity has been detected, either in the expectant mother, the family members, or the child, the clinician must form a multidisciplinary team that aims to offer integrated therapy possibilities, based on needs. The latter, initiated at the right time, can reduce the need to use pharmacotherapy or radical surgical therapy. To achieve optimal results, it is essential to incorporate psychological and nutritional therapy, as well as lifestyle adjustment instructions, that both the patient and their family must adhere to.

Future research directions: To enhance adjuvant therapy for childhood obesity and to deepen the understanding of its multifactorial etiology, future studies should focus on standardizing and integrating alternative, non-pharmacological approaches that modulate the internal microenvironment (e.g., counteracting intestinal dysbiosis). Further investigations are warranted to evaluate the long-term effectiveness of personalized, multidisciplinary interventions, as well as to develop evidence-based guidelines for the implementation of preventive and therapeutic strategies at both individual and population levels.

## Figures and Tables

**Table 1:** The main characteristics of the most common genetic imbalances implicated in childhood obesity [38,40,43,44].

Gena	Function	Role	Observation
*LEP*	- modulates the activity of presynaptic GABAergic neurons by inhibiting the NPY/AgRP pathway	- decreases appetite and increases energy expenditure	- secreted by white adipose tissue - encoded by a gene on chromosome 7 - receptors are found in the central nervous system and peripheral organs (liver, skeletal muscles, pancreatic beta cells, and fat cells)
*POMC*	- modulates the leptin-melanocortin system - *POMC* deficiency causes the absence of ACTH and alpha-MSH	- appetite-suppressing gene - deficiency-hyperphagia, lower resting metabolic rate, and severe obesity, red hair and pale skin	- location: chromosome 2
*MC4R*	- modulates the activity of leptin and growth hormone	- influences energy homeostasis and food intake behavior - may be accompanied by severe hyperinsulinemia	- clinical features: “big child” with - excess fat and reduced muscle mass - complications: sleep apnea
*FTO*	- encodes a 2-oxoglutarate-dependent nucleic acid demethylase overexpressed in the hypothalamic nuclei involved in modulating energy balance	- there are associations of single-nucleotide polymorphisms of the *FTO* gene and increased intake of dietary fat, protein, energy, increased appetite, and decreased satiety - *FTO* SNPs also influence the expression of other genes	- location: chromosome 16q12.2

*LEP*: leptin, *POMC*: proopiomelanocortin, *MC4R*: melanocortin-4 receptor, *FTO*: fat mass and obesity associated gene, GABA: gamma-aminobutyric acid, ACTH: adrenocorticotropic hormone, MSH: melanocyte-stimulating hormone, NPY: neuropeptide Y, AgRP: agouti-related protein, SNP: single-nucleotide polymorphism.

**Table 2:** Vulnerable points in the emergence and maintenance of childhood obesity.

Classification	Factor	Countering Opportunities
Antenatal	- Genetic defects - Maternal metabolic health	1. Genetic tests 2. Development of targeted therapies 3. Antenatal genetic advice 4. Scheduling/anticipating the pregnancy 5. Monitoring of maternal health and nutrition
Perinatal	- Type of birth (natural/caesarean section) - Gestational age - Birth weight - Antibiotherapy - Type of feeding (breastfeeding/feeding with milk formula) - Breastfeeding program - The moment of introducing complementary food	1. Promotion of natural birth where caesarean section is not a medical indication 2. Discouraging the use of excessive antibiotic therapy in mother and child 3. Promotion of breastfeeding up to one year, alternative in cases with medical indication: protein-balanced mixed nutrition 4. Promotion of the breastfeeding program 5. Introduction of complementary food between 17–26 weeks 6. Currently, the practice of receptive feeding of infants is encouraged
Postnatal	Precarious socio-economic status Intrafamilial influence Religious considerations Unhealthy eating habits Sedentary behavior Prolonged time spent in front of screens Excessive parental control Characteristics of urban settlements (pollution, traffic, noise) Sleep deprivation Stressors Food marketing through social applications Psycho-somatic disorders Improper use of pharmacotherapy (e.g., psychostimulants, neuroleptics, antidepressants) Endocrinological imbalances	1. Programming of nutritional education in the population at risk 2. Promotion of balanced breakfast and family meals; 3. Combating obesogenic characteristics perpetuated transgenerationally 4. Promoting an active lifestyle 5. Creating communities with healthy life principles 6. Promotion of mixed neighborhood grocery stores 7. Fighting pollution, heavy traffic and noise 8. Effective rest program 9. Elimination of stressful factors 10. Psychotherapy and psychiatric monitoring of the patient 11. Adaptation of medication doses 12. Endocrinopathies screening in the case of the association of suggestive clinical characteristics

**Table 3:** Synthesis of molecular and genetic mechanisms involved in the predisposition and progression of obesity in children: recent data and translational perspectives.

Molecular Mechanism	Recent Scientific Achievements	Representative Studies/Reviews
Polygenic genetics and associated variants	Identification of over 1100 genetic loci associated with obesity and body composition traits; studies exploring the role of SNPs and CNVs in the regulation of BMI and response to lifestyle interventions.	[147]
Epigenetics and DNA methylation	Emerging evidence on specific methylation sites associated with BMI from birth to adolescence, as well as a meta-analytic examination of EWAS for childhood obesity.	[148]
Epigenome changes in pediatric obesity	Recent study highlights how epigenetic marks (including DNA methylation and microRNA) are linked to obesity phenotypes in children, drawing attention to the need for more research on histones and chromatin remodeling proteins.	[149]
Intergenerational transmission of risk	Evidence supporting that obesity in parents (maternal/paternal) can influence the risk of obesity in offspring through epigenetic mechanisms and other biological processes programmed during the perinatal period.	[150]
Effects of maternal obesity on the genome of offspring	Animal model showing that maternal obesity induces genomic hypermethylation in oocytes and can be transmitted to offspring, and melatonin moderates DNMTs expression.	[151]
The role of microRNA and ncRNA in childhood	Findings of differentially expressed microRNAs (e.g., miR 15b 5p, miR 486 5p, hsa miR 122 5p) associated with pediatric obesity, suggesting that post-translational transcriptional regulation is an important mechanism.	[149]
Rare and monogenic forms of obesity	Comprehensive review of monogenic forms of childhood obesity—usually severe, with defects in the leptin-melanocortin pathways that regulate hunger and satiety.	[152]
Gene-environment interactions and molecular networks	Recent analyses highlight how environmental factors (hypercaloric diets, sedentary lifestyle) affect the epigenome (methylation, histone modifications) and gene expression related to metabolism.	[153]

## Data Availability

No new data were generated.
